# Editorial: Pharmacokinetic-pharmacodynamic model of drugs and their pharmacokinetic differences between normal and disease states

**DOI:** 10.3389/fphar.2024.1451176

**Published:** 2024-08-05

**Authors:** Zipeng Gong, Ruixing Chen, Ling Ye, Guo Ma, Yanfang Xian, Kaustubh Kulkarni

**Affiliations:** ^1^ State Key Laboratory of Functions and Applications of Medicinal Plants, Guizhou Provincial Key Laboratory of Pharmaceutics, School of Pharmacy, Guizhou Medical University, Guiyang, China; ^2^ Key Laboratory of Basic Pharmacology of Ministry of Education, Zunyi Medical University, Zunyi, China; ^3^ Guizhou Provincial Engineering Research Center for the Development and Application of Ethnic Medicine and TCM, Guizhou Medical University, Guiyang, China; ^4^ NMPA Key Laboratory for Research and Evaluation of Drug Metabolism, Guangdong Provincial Key Laboratory of New Drug Screening, School of Pharmaceutical Sciences, Southern Medical University, Guangzhou, China; ^5^ School of Pharmacy, Fudan University, Shanghai, China; ^6^ School of Chinese Medicine, Faculty of Medicine, The Chinese University of Hong Kong, Shatin, Hong Kong SAR, China; ^7^ Boundless Bio Inc, San Diego, CA, United States

**Keywords:** normal status, disease states, metabolizing enzymes, transporters, pharmacokinetic-pharmacodynamic link model

This Research Topic is part of a series with “Pharmacokinetic Differences of Drugs and Their Regulatory Mechanisms Under Dual Status Including Normal and Diseased Organism”

In the main frame of the above Research Topic, 16 contributions have been published including but not limited to pharmacokinetic comparisons between normal and disease status, the changes in the expression and function of drug-metabolizing enzymes and drug transporters in disease status and their associated regulatory mechanisms, ADME/toxicity of drugs as well as their modulation, drug-drug interactions mediated by nuclear receptors, transporters and metabolic enzymes based on the approaches and techniques of pharmacokinetics, transcriptomics and metabolomics (Gong et al., 2022). However, pharmacokinetic studies are not only essential for new drug development, but the study of pharmacokinetic-pharmacodynamic (PK-PD) models also plays a significant role in this process. The PK-PD model can uncover the intrinsic relationship between drug concentration and its effects, assisting in understanding the dynamic characteristics of the drug’s action site within the body. It helps deduce the action site producing the effect and the drug concentration at that site, enabling researchers to grasp the comprehensive characteristics of the drug’s pharmacokinetic and pharmacodynamic processes *in vivo* (Derendorf et al., 2000)However, preclinical pharmacokinetic data are mainly derived from healthy animals, which is unreasonable. On one hand, patients are the ultimate users of drugs, as drugs are primarily used to treat individuals with diseases (Liang et al., 2023). On the other hand, the body’s pathological state and the severity of the disease significantly impact the ADME (absorption, distribution, metabolism, and excretion) of drugs, which are crucial for determining the safety and efficacy of clinical treatments. Consequently, studying pharmacokinetics and the PK-PD model in disease states is more meaningful and relevant to clinical practice.

Considering that drugs are primarily used in the body under disease conditions, this research will not only continue to focus on the pharmacokinetic differences of drugs, including Traditional Chinese medicine, and their regulatory mechanisms under both normal and diseased states, but will also emphasize the study of drug PK-PD models based on cellular pharmacokinetics and integrated pharmacokinetics. We aim to provide references and insights for individualized, safe, and effective drug use, and for optimizing the drug evaluation system based on disease states (Gong et al., 2015).

Within the framework of this Research Topic, 14 contributions have been published, covering subjects including, but not limited to: the pharmacokinetic differences of drugs, including Traditional Chinese medicine, and their mechanisms under normal and diseased states, the study of cellular pharmacokinetics, the changes in the expression and function of drug-metabolizing enzymes and drug transporters in disease status and their related regulatory mechanisms, the research on intestinal microbiome mediated drug metabolism and its effect on drug efficacy and toxicity, ADME/toxicity of drugs as well as their regulation, drug-drug interactions mediated by nuclear receptors, transporters and metabolic enzymes based on the methods and techniques of pharmacokinetics, transcriptomics, and metabolomics, the study of drug PK-PD model based on the cellular pharmacokinetics and integrated pharmacokinetics.

Nwabufo et al. demonstrated the dysregulated expression of 25 clinically relevant drug metabolizing enzymes and membrane transporters (DMETs) in Vero E6 cells and post-mortem lung tissues of COVID-19 patients. They found that SARS-CoV-2 infection leads to DMETs dysregulation, with inflammatory cells being the main drivers of the differences in DMETs localization between COVID-19 and control lung tissues.

Nie et al. established a two-compartment population pharmacokinetics (PopPK) modelfor naloxone in adult patients undergoing general anesthesia. Compared the pharmacokinetic discrepancy of naloxone between covariates and dosage regimens in 47 patientsby Monte Carlo simulations, and the conclusion was that fixed-dose regimens were better.

Lian et al. elucidated the mechanism by which the compatible herbs in the Jin-Gu-Lian formula reduce Alangium chinense (AC)-induced neurotoxicity. The combined use of AC and the herbal extract of the Jin-Gu-Lian formula (CH) significantly reduced the plasma exposure levels of two main components of AC, and markedly downregulated the gene expression of cytochrome P450 enzymes induced by AC.

Xiong et al. developed a highly efficient method for simultaneously determining four major compounds in the Mori Cortex total flavonoid extract. Thus, they studied the pharmacokinetic differences between normal and diabetic rats after oral administration, providing a pharmacokinetic basis for the pharmacological and toxicological studies of Mori Cortex.

Nie et al. compared the pharmacokinetic differences of seven active components of Ling-Gui-Zhu-Gan decoction (LGZGD) in normal rats and rats with non-alcoholic fatty liver disease (NAFLD). They found that the expression levels of the metabolic enzyme UGT1A1 and nine transport proteins were significantly reduced in NAFLD rats, which might explain the pharmacokinetic differences between normal and NAFLD rats.

Wang et al. explored the pharmacokinetic differences caused by metabolic enzymes for six main components of Huangqi Liuyi Decoction in mice liver microsomes. The metabolism of the active components might be related to CYP2C37, CYP2C11, CYP1A2, CYP2E1, and CYP3A11.

Liu et al. investigated the toxicity and pharmacodynamic components of Asarum essential oil (AEO) using molecular distillation technology. They identified the main anti-inflammatory components as methyl eugenol, α-pinene, and β-pinene, and the main toxic components as camphor, methyl eugenol, and 3,5-dimethoxytoluene.

Zhan et al. found that the inhibitory effect of vortioxetine on CYP450 enzymes is reversible and lacks time-dependent inhibition. Additionally, they identified the types of inhibition mechanisms of vortioxetine on CYP450 enzymes in human and rat liver microsomes. This study provides important guidance for the safe clinical use of vortioxetine.

Alasmari et al. used the PBPK model to accurately simulate the pharmacokinetic differences of ceftriaxone in healthy individuals and patients with mild, moderate, and severe chronic kidney disease (CKD). Additionally, they found that dose reduction could achieve same exposure comparable to that of healthy individuals.

Ye et al. elucidated the characteristics and pharmacological effects of Glycycoumarin (GCM) through pharmacokinetic studies. They found that GCM was rapidly absorbed and converted into its conjugated metabolites with low bioavailability after administration. Additionally, it was distributed in various tissues except the brain.

Sun et al. developed a simple and efficientmethod to determine the concentration of riluzole in human plasma, which successfully applied it to the pharmacokinetic study of riluzole in Chinese patients with amyotrophic lateral sclerosis (ALS).

Tao et al. compared the PK, PD, and safety of Ciprofol in Chinese patients with normal renal function and those with impaired renal function after injection. Their investigation revealed that Ciprofol was well tolerated, providing an important guide to clinical dose adjustment.

Chen et al. found that the interaction between lacosamide and nisoldipine significantly altered the pharmacokinetic parameters of lacosamide and its metabolites *in vivo* and *in vitro*. In addition, the mechanism of nisoldipine and lacosamide metabolism was associated with mixed inhibition.

Zhu et al. studied the pharmacokinetics of 15 prototype components in the extract of Pogostemon cablin (PC) after oral administration in rats. It providing reference value for the clinical application of PC.

Therefore, given that drugs are largely taken by patients, the Research Topic focused mainly on PK-PD Model of drugs and their pharmacokinetic differences between normal and disease states, which could optimize the design of rational dosing regimens for clinical therapeutics, and provide reference individualized safety and effective drug use.
